# Macadamia (*Macadamia integrifolia*) Oil Prevents High-Fat Diet-Induced Lipid Accumulation and Oxidative Stress by Activating the AMPK/Nrf2 Pathway

**DOI:** 10.3390/foods13223672

**Published:** 2024-11-18

**Authors:** Ming Zhang, Yuhan Zhang, Lingdong Li, Changbin Wei, Taotao Dai, Ya Li, Xixiang Shuai, Liqing Du

**Affiliations:** 1 South Subtropical Crop Research Institute, China Academy of Tropical Agricultural Sciences, Zhanjiang 524091, China; zhangmingqau@163.com (M.Z.); lingdong0510@163.com (L.L.); zbwcb@163.com (C.W.); liya1995ncu@163.com (Y.L.); shuaixixiang1989@163.com (X.S.); 2 Key Laboratory of Tropical Fruit Biology, Ministry of Agriculture & Rural Affairs, Key Laboratory of Hainan Province for Postharvest Physiology and Technology of Tropical Horticultural Products, Zhanjiang 524091, China; nyszhangyh@163.com; 3 State Key Laboratory of Food Science and Technology, Nanchang University, Nanchang 330047, China; ncubamboo@163.com

**Keywords:** macadamia oil, high-fat diet, oxidative stress, lipid lowering, AMPK/Nrf2 pathway

## Abstract

Hyperlipidemia, characterized by an abnormal lipid metabolism, is related to multiple cardiovascular diseases that pose challenges to global public health. Macadamia oil (MO), rich in monounsaturated fatty acids (around 80%), is regarded as a functional oil used to regulate lipid accumulation. Nonetheless, the lipid-lowering mechanism of MO is still unknown. Therefore, the lipid-lowering effects of MO in high-fat diet (HFD)-induced hyperlipidemic mice were evaluated in this study. The results revealed that MO could effectively reduce body weight and the organ index and improve serum lipid levels by reducing total cholesterol, triglycerides, and low-density lipoprotein cholesterol levels and elevating high-density lipoprotein cholesterol levels. Additionally, MO supplementation could improve abnormal liver function caused by hyperlipemia, characterized by decreased liver enzyme levels, including alanine aminotransferase and aspartate aminotransferase. Meanwhile, MO also exhibited an inhibitory effect on oxidative stress and lipid accumulation caused by an HFD. Moreover, findings from qRT-PCR and Western blotting analyses suggest that MO supplementation markedly prevented hyperlipidemia by inhibiting the expression of AMPK pathway-related genes, SREBP-1c, FAS, ACC, and PPAR-γ, as well as upregulating the levels of Nrf2, HO-1, and γ-GCS. These results indicate that MO attenuates lipid accumulation in vivo via AMPK/Nrf2 pathway activation, suggesting that MO could serve as a dietary supplementation or medication for treating hyperlipidemia.

## 1. Introduction

Hyperlipidemia is a primary risk factor for cardiovascular disease, which commonly manifests in abnormal lipid metabolism and liver function [1]. Meanwhile, hyperlipidemia has been demonstrated to relate to many common human diseases, such as stroke, hypertension, and coronary heart disease [2]. Notably, with the rapid development of the economy, high-fat and low-fiber diets have gradually become a favored dietary pattern, which has led to hyperlipidemia emerging as a serious global public health problem [3]. Currently, several drugs, including statins, fibrates, and niacin, have been verified to be effective in alleviating hyperlipidemia. Unfortunately, long-term exposure to these drugs may cause some side effects, such as liver injury, muscle pain, and dyspepsia [4]. There is no doubt that exploring new medications and dietary supplements is crucial for replacing chemicals to regulate lipid metabolism.

Over the past decades, scientists have made great efforts to find new therapeutic methods to lower lipid levels. Excitingly, a plethora of studies have highlighted the potential of using natural products to treat hyperlipidemia, such as polyphenols, polysaccharides, fish oil, and vegetable oils [5,6,7]. Surprisingly, it has been confirmed that the side effects of natural products are much milder and safer in contrast to synthetic drugs [8]. Meanwhile, growing evidence shows that the administration of vegetable oils (e.g., olive oil and rapeseed oil) with high monounsaturated fatty acids (MUFAs) content could effectively attenuate lipid accumulation by reducing serum total cholesterol (TC), triglyceride (TG), low-density lipoprotein cholesterol (LDL-C), alanine aminotransferase (ALT), and aspartate aminotransferase (AST) levels in addition to elevating high-density lipoprotein cholesterol (HDL-C) [9,10]. Macadamia oil (MO), obtained from *Macadamia integrifolia* seeds, has been proven to be rich in MUFAs (approximately 64% oleic acid and 16% palmitoleic acid). It offers various biological benefits, including alleviating cardiovascular disease and anti-hyperlipidemic, anti-tumor, and anti-inflammatory properties [11,12]. Nevertheless, the effects of MO in lowering lipid levels in hyperlipidemic mice induced with a high-fat diet (HFD) have yet to be characterized, and the underlying mechanism of action remains unclear.

Consequently, a hyperlipidemic mice model induced with an HFD was established in the present study. Then, the effects of MO administration (0.11, 0.22, and 0.33 mL/20 g of body weight (BW)) on BW, organ index, serum lipids, liver enzymes, oxidative stress, and the morphology of liver and adipose tissues were investigated to explore the effect of MO in lipid accumulation. Moreover, quantitative real-time polymerase chain reaction (qRT-PCR) and Western blotting (WB) were employed to analyze the expression of adenosine 5′-monophosphate activated protein kinase (AMPK) and nuclear red cell 2 associated factor (Nrf2) pathway-related genes and proteins to explore the anti-hyperlipidemic mechanism of MO. This work will provide strong scientific evidence for the advanced application of MO in multiple functional foods and medications.

## 2. Materials and Methods

### 2.1. Materials and Chemicals

Macadamia nuts of the variety 791 were collected from the macadamia planting base of the Lincang Academy of Forestry (Lincang City, China) in September 2023. Simvastatin, phosphate-buffered saline (PBS), 2′,7′-dichlorofluorescin diacetate (DCFH-DA), hematoxylin, eosin Y, and 4% paraformaldehyde fixative were purchased from Solarbio Co., Ltd. (Beijing, China). Reactive oxygen species (ROS), AMPK, p-AMPK, sterol regulatory element binding protein-1c (SREBP-1c), acetyl-CoA carboxylase (ACC), fatty acid synthetase (FAS), peroxide-object proliferators γ (PPAR-γ), heme oxygenase-1 (HO-1), γ-glutamylcysteine synthetase (γ-GCS), nuclear red cell 2 associated factor (Nrf2), and glyceraldehyde-3-phosphate dehydrogenase (GAPDH) were provided by Beyotime Biotechnology Co., Ltd. (Shanghai, China). All chemicals and reagents utilized in this experiment were of analytical grade.

### 2.2. Preparation of MO

MO was produced by the cold press method according to a previous study [13]. In brief, the collected fresh macadamia nuts were peeled, dried, shelled, and pressed with a D-40 screw oil press (Zhongshan Romeo Seiko Equipment Co., Ltd., Zhongshan, China). And the pressed oil was centrifuged at 3840× *g* for 20 min using an HR/T20 MM laboratory centrifuge (Hunan Herexi Instrument Co., Ltd., Changsha, China) to remove solid impurities and obtain clear MO. Finally, MO was stored in a food-grade plastic bottle at 4 °C for the following experiments.

### 2.3. Animal Experimental Design

**Figure 1:**
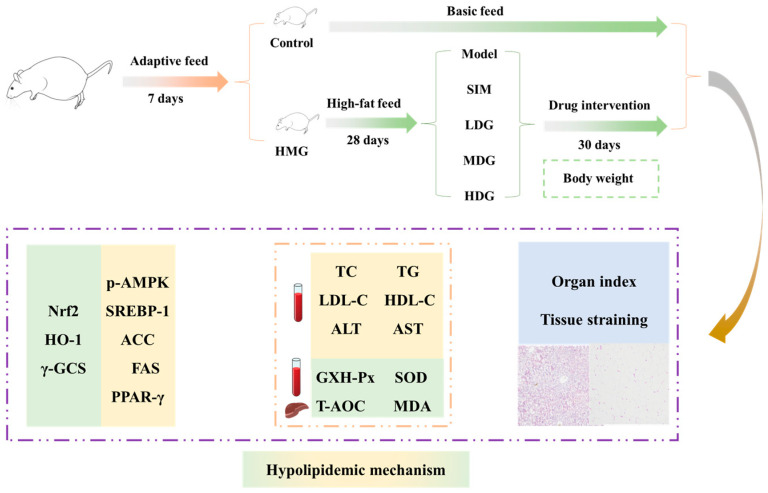
The diagram of the timeline for the design of the animal experiments.

In the present study, animal experiments (Figure 1) were conducted in male-specific pathogen-free Kunming mice (license number, SCXK 2019-0010; Sipeifu Biotechnology Co., Ltd., Beijing, China) with an original BW of 30–32 g. Before the experiments, the mice underwent adaptive feeding for 7 days with basic feed (Jiangsu Xietong Biological Co., Ltd., Nanjing, China), having free access to water in an environment of 23 ± 2 °C, with 50 ± 10% relative humidity, and a natural light/dark cycle. Two groups were designated: a control group (Control) and a hyperlipidemic model group (HMG). The Control continued to be fed basic feed, while the HMG was fed high-fat feed (Jiangsu Xietong Biological Co., Ltd., Nanjing, China). Subsequently, the HMG was randomly divided into five experimental groups after feeding for 28 days, including the model group (Model, HFD), simvastatin-treated group (SIM, 10 mg simvastatin/kg of BW), and low-, medium-, and high-dose MO-treated groups (LDG, MDG, and HDG, respectively; 0.11, 0.22, and 0.33 mL MO/20 g of BW, respectively). During the drug intervention experiments (30 days), the BW of all the experimental mice was recorded every 5 days.

After the final dose of the drug, each group was fasted overnight. Whole blood was collected via blood collection from the eyes the following day and then centrifuged at 865× *g* for 15 min at 4 °C to separate serum for the follow-up evaluation. Meanwhile, the liver, kidney, spleen, and testicular adipose tissue were immediately collected, rinsed, and weighed. The organ index was calculated using Equation (1). Then, the liver and testicular adipose tissue were photographed, and one part was stored in a fixative solution of 4% paraformaldehyde, while the others were frozen at −80 °C for the following determination.

(1) $$\mathrm{Organ}\;\mathrm{index}=\frac{\mathrm{Tissue}\;\mathrm{weight}}{\mathrm{Body}\;\mathrm{weight}}\times 100\%$$

### 2.4. Biochemical Analysis of Serum and Liver

#### 2.4.1. Determination of Serum Biochemical Indicators

The serum lipid levels of TC, HDL-C, TG, and LDL-C and the serum liver enzyme levels of ALT and AST levels were detected using commercially available kits according to their instructions.

#### 2.4.2. Determination of ROS in Liver

The ROS levels in the liver were analyzed following the method by Zeng et al. [14] with suitable modifications. In brief, the liver tissue was sliced and resuspended using the PBS solution. Then, the separated liver cells were washed and incubated using 1 mL of a DCFH-DA diluted solution for 20 min at 37 °C. Finally, the liver cell suspension was washed using a serum-free medium and then resuspended with 500 µL PBS solution. The fluorescence was evaluated by a cytoFLEX flow cytometer (Beckman coulter, Brea, CA, USA).

#### 2.4.3. Evaluation of Oxidative Stress Indicators in Serum and Liver

During this process, 1.0 g of liver tissue was weighed and then homogenized with 9 mL of ice-cold PBS solution. Subsequently, the liver sample was centrifuged at 865× *g* for 15 min. Finally, the superoxide dismutase (SOD), catalase glutathione (GSH-Px), total antioxidant capacity (T-AOC), and methane dicarboxylic aldehyde (MDA) levels of the liver and serum (prepared in Section 2.3) were analyzed using commercially available kits according to their instructions.

### 2.5. Morphological Observation of Liver and Testicular Adipose Tissue

#### 2.5.1. Hematoxylin/Eosin (HE) Staining of the Testicular Adipose Tissue

The HE staining process was conducted according to the method of Liu et al. [15] with suitable modifications. At first, testicular adipose tissue was subjected to dehydration, transparency, wax immersion, and embedding. Specifically, the tissues were dehydrated in 75%, 85%, 90%, 95%, and 100% ethanol/water solutions for 4.0, 2.0, 1.5, 1.0, and 0.5 h, respectively. Then, the tissues were treated with an anhydrous ethanol and xylene mixture (1:1, *v*/*v*) for 10 min, followed by submersion twice in xylene for 10 min each time, while the wax immersion process was repeated three times at 60 °C. After embedding, the sections were conducted using an RM 2016 rotary microtome (Leica Co., Ltd., Shanghai, China). For the dewaxing process, the paraffin sections were treated with xylene three times for 20 min each and soaked in 100%, 90%, 80%, and 70% ethanol/water solutions and distilled water for 5 min, respectively. The tissues were stained with HE before being dehydrated and immersed in xylene. At last, the sections were naturally dried in air and sealed with neutral gum for observation under an Fj3 microscope (Nikon Co., Ltd., Shanghai, China).

#### 2.5.2. Oil Red O Staining of Liver Tissue

The Oil Red O staining process of liver tissue was conducted according to a previous study by He et al. [16] with suitable modifications. In brief, the frozen liver tissue was sectioned, rewarmed to room temperature, and rinsed with distilled water followed by immersion in a 60% isopropyl alcohol/water solution before being stained with the Oil Red O solution for 10 min. The slices were then differentiated with a 60% isopropyl alcohol/water solution before being counterstained with hematoxylin for 5 min. Finally, the stained section was sealed using a water-soluble mounting medium for further observation.

### 2.6. Quantitative Real-Time Polymerase Chain Reaction (qRT-PCR) Analysis

The qRT-PCR assay was conducted using the method by Chu et al. [17] with suitable modifications. In brief, the total RNA from the liver tissue was isolated using a Trizol solution (Ambion, Austin, TX, USA). Then, 3 µg of isolated total RNA was weighed to reverse-transcribe into cDNA using HiScript^®^ II Q RT SuperMix for qPCR (+gDNA wiper) (Vazyme, Nanjing, China). During this process, the reaction was performed at 50 °C for 15 min, 85 °C for 5 s, and 4 °C for 10 min. Next, the cDNA was amplified using specific primers (Table 1), and the target gene expressions were determined using the QuantStudio 6 real-time PCR system (ABI, Carlsbad, CA, USA).

### 2.7. WB Analysis

The experiments were carried out based on a methodology from a previous study by Chen et al. [18]. The liver tissue was homogenized using a CEBO-24 homogenizer (Cebo Biotechnology Co., Ltd., Shanghai, China) with 200 µL of lysate buffer containing 2 µL of phenyl methane sulfonyl fluoride and 2 µL of phosphatase inhibitor and centrifuged at 13,840× *g* for 5 min after standing for 30 min at 4 °C. Meanwhile, the protein of the supernatant was separated using sodium dodecyl sulfate-polyacrylamide gel electrophoresis, and the target strips were cut according to the marker strip. It was then transferred to a polyvinylidene fluoride (PVDF) membrane (0.45 µm) before being immersed in a 5% bovine serum albumin standard solution (*w*/*v*) for 2 h. Subsequently, SREBP-1c, p-MARK, Nrf2, HO-1, ACC, FAS, γ-GCS, PPAR-γ, and GAPDH were added and incubated for 12 h at 4 °C. Next, the PVDF membrane was washed with tris-buffered saline (0.1% Tween-20, 3 g/L trimethylol aminomethane, and 3 g/L NaCl) and then incubated with the secondary antibody for 2 h, followed by rewashing. Finally, the PVDF membrane was incubated with the enhanced chemiluminescence solution for further observation.

### 2.8. Statistical Analysis

The animal and in vivo experiments were carried out at least eight and three times, respectively. The experimental data were analyzed by GraphPad Prism 8.2.1 (San Diego, CA, USA) and expressed as mean value ± standard deviation. Statistical significance was determined using one-way analysis of variance and *t*-tests, with *p* < 0.05 considered statistically significant.

## 3. Results and Discussion

### 3.1. Effects of MO on BW and Organ Index

**Figure 2:**
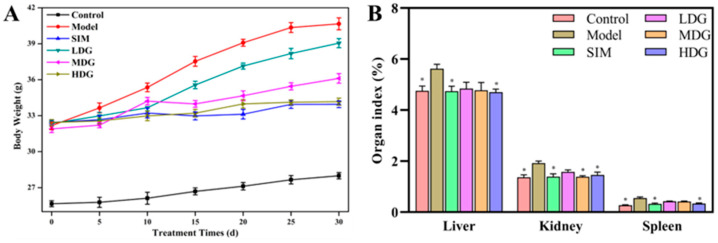
Body weight and organ index changes in hyperlipidemic mice after 30 days of macadamia oil intervention. (**A**) Body weight. (**B**) Organ index. * indicates *p* < 0.05 versus the Model.

Long-term HFD can induce abnormal lipid accumulation, resulting in weight gain and organ enlargement [6]. Changes in the BW of the SIM- and MO-treated groups are shown in Figure 2A. The Model exhibited a more significant increase in BW than the Control (*p* < 0.05), and the weight gain rate of the SIM- and MO-treated groups was clearly inhibited relative to the Model. The BW showed a reduction of 4.0%, 11.2%, 16.0%, and 16.5% in the LDG, MDG, HDG, and SIM in comparison with the Model, respectively. Furthermore, the BW showed a dose-dependent decrease in MO, with the high-dose MO exhibiting a comparable weight loss similar to that of simvastatin. These findings were similar to previous reports on flaxseed oil [19] and fish oil [7] supplementation. In simple terms, MO could effectively inhibit weight gain induced by an HFD.

Generally, changes in organs are often evaluated by the organ index. As depicted in Figure 2B, the liver index for the LDG, MDG, and HDG was lower compared to the Model group. Specifically, only the HDG exhibited significant differences in the liver index compared with the Model (*p* < 0.05), whereas no significant differences were observed in the LDG or MDG relative to the Model group. Meanwhile, the liver index of the HDG and SIM showed no significant difference (*p* < 0.05), and the kidney and spleen indices also showed a similar effect. Additionally, excessive organ index values were known to be indicators of diseases such as inflammation and hypolipidemia [20,21], indicating that MO could be used as a drug or healthcare product to improve health.

### 3.2. Effects of MO on Serum Biochemical Indicators

**Figure 3:**
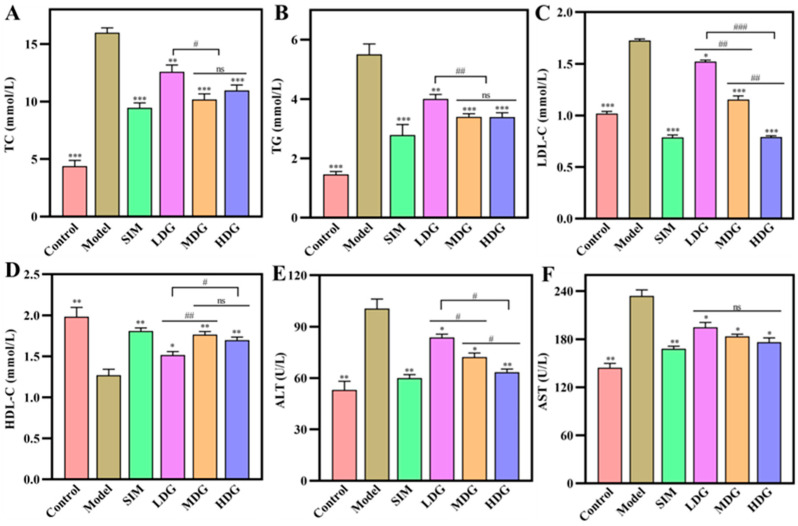
Serum lipids and serum liver enzymes changes in hyperlipidemic mice after 30 days of macadamia oil intervention. (**A**–**D**) Serum TC, TG, LDL-C, and HDL-C levels; (**E**,**F**) serum ALT and AST levels. ns denotes no significant differences between different letters at *p* < 0.05. *, **, and *** indicate *p* < 0.05, *p* < 0.01, and *p* < 0.001 versus the Model, respectively. #, ##, and ### denote significant differences between different letters at *p* < 0.05, *p* < 0.01, and *p* < 0.001, respectively.

Lipid metabolism disorders typically manifest as symptoms of hyperlipidemia, including elevated serum TC, TG, and LDL-C levels and reduced serum HDL-C levels. Moreover, hyperlipidemia is usually accompanied by fatty liver and liver damage, characterized by an increase in serum ALT and AST levels. In this study (Figure 3), the serum TC, TG, LDL-C, ALT, and AST levels of the Model were significantly higher than those of the Control. The opposite results were found in the HDL-C levels, suggesting that a hyperlipidemic mice model was successfully established. Lipid metabolism and the liver were disrupted by the administration of an HFD.

Figure 3A indicated that serum TC levels of the MO-treated groups were significantly reduced after 30 days of MO intervention compared with the Model (*p* < 0.05). The serum TC levels of the LDG, MDG, and HDG decreased by 21.2%, 36.2%, and 31.4% compared with that of the Model, respectively, suggesting that medium-dose MO was more effective in reducing the serum TC levels, which was comparable to the simvastatin. These results were aligned with the study by Zeng et al. [22], who suggested that medium-dose *Prunella vulgaris* seed oil (500 mg/kg BW) has a better effect in reducing serum TC levels than low-dose and high-dose treatments (200 and 800 mg/kg BW, respectively). Similarly, the medium-dose MO exhibited a more significant reduction in the serum TG, ALT, and AST levels (Figure 3B,E,F) compared to the other doses, suggesting that MO contributed to the improvement in lipid metabolism and liver protection. Additionally, wheat bran oil [23] and flaxseed oil [19] supplementation also exhibited significant intervention effects on serum TC and TG levels.

HDL-C serves as an endogenous cholesterol ester, facilitating the transfer of excessive cholesterol from the blood to the liver to reduce serum cholesterol levels. Thus, HDL-C is an important index of cholesterol metabolism. Figure 3C depicted the HDL-C levels of all the experimental groups. The HDL-C levels of the LDG, MDG, and HDG were increased by 16.2%, 38.8%, and 33.5%, respectively, in comparison with the Model. The LDL-C levels (Figure 3D) exhibited a significant decrease as the MO dose increased (*p* < 0.05). Meanwhile, the MDG, HDG, and SIM showed no significant differences in HDL-C levels (*p* < 0.05). It was to say that MO could alleviate hyperlipidemia by increasing HDL-C levels and decreasing LDL-C levels. These results were in line with the study by Gao et al. [6], who evaluated the inhibitory effects of sea buckthorn fruit oil on serum lipids. Importantly, a reduction in serum LDL-C levels is beneficial for preventing atherosclerosis because oxidized LDL can promote the lipid uptake of macrophages [24]. Additionally, some bioactive components, such as polysaccharides [17] and polyphenols [25], also demonstrated effectiveness in serum lipid level intervening.

### 3.3. The Effects of MO on ROS in the Liver

**Figure 4:**
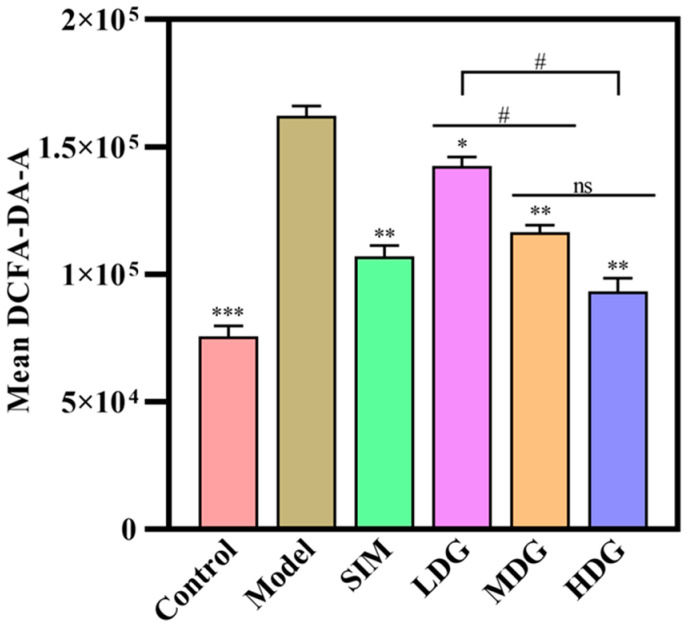
Reactive oxygen species changes in liver tissue after 30 days of macadamia oil intervention. ns denotes no significant differences between different letters at *p* < 0.05. *, **, and *** indicate *p* < 0.05, *p* < 0.01, and *p* < 0.001 versus the Model, respectively. # denotes *p* < 0.05 versus the LDG.

An HFD can easily lead to lipid accumulation in the liver, producing excessive ROS. However, ROS accumulation can lead to oxidative damage in mitochondria and cell membranes, resulting in the release of multiple enzymes and ultimately causing cell death [14]. Hence, the ROS levels in the liver were analyzed, and the results are shown in Figure 4. The ROS levels in the Model exhibited a significant increase in comparison with the Control, suggesting that an HFD induced oxidative damage. Moreover, it was observed that simvastatin and MO treatment significantly inhibited ROS generation compared to the Model. Furthermore, the high-dose MO treatment demonstrated the most significant inhibition of ROS, with a decrease of 42.50% in comparison with the Model. This was followed by the medium-dose and low-dose treatments, which decreased the ROS levels by 28.19%, and 12.07%, respectively. Moreover, these results were aligned with the findings of Feng et al. [26], who demonstrated that pumpkin seed oil could effectively decrease ROS levels and verified that the antioxidant properties of vegetable oil were positively correlated to MUFAs content. However, nigella seed oil [27] could not prevent ROS overproduction induced by 7-ketocholesterol and 24S-hydroxycholesterol, which might be attributed to its low content of MUFAs (40.04%). Nevertheless, it was reported that oleic acid (C18:1 n-9) could attenuate the overproduction of ROS [28], which might contribute to the significant inhibitory effect of MO on ROS generation.

### 3.4. The Effects of MO on Oxidative Stress Indicators in the Serum and Liver

An HFD had been confirmed to be responsible for increasing oxidative stress in organs, which might result in several physical degenerative diseases. Therefore, the levels of oxidative stress indicators such as GSH-Px, SOD, T-AOC, and MDA were evaluated in the present study. In particular, the GSH-Px, SOD, and T-AOC function as antioxidant enzymes responsible for scavenging free radicals [29]. As a product of lipid oxidation, MDA usually represents the degree of lipid peroxidation [6].

**Figure 5:**
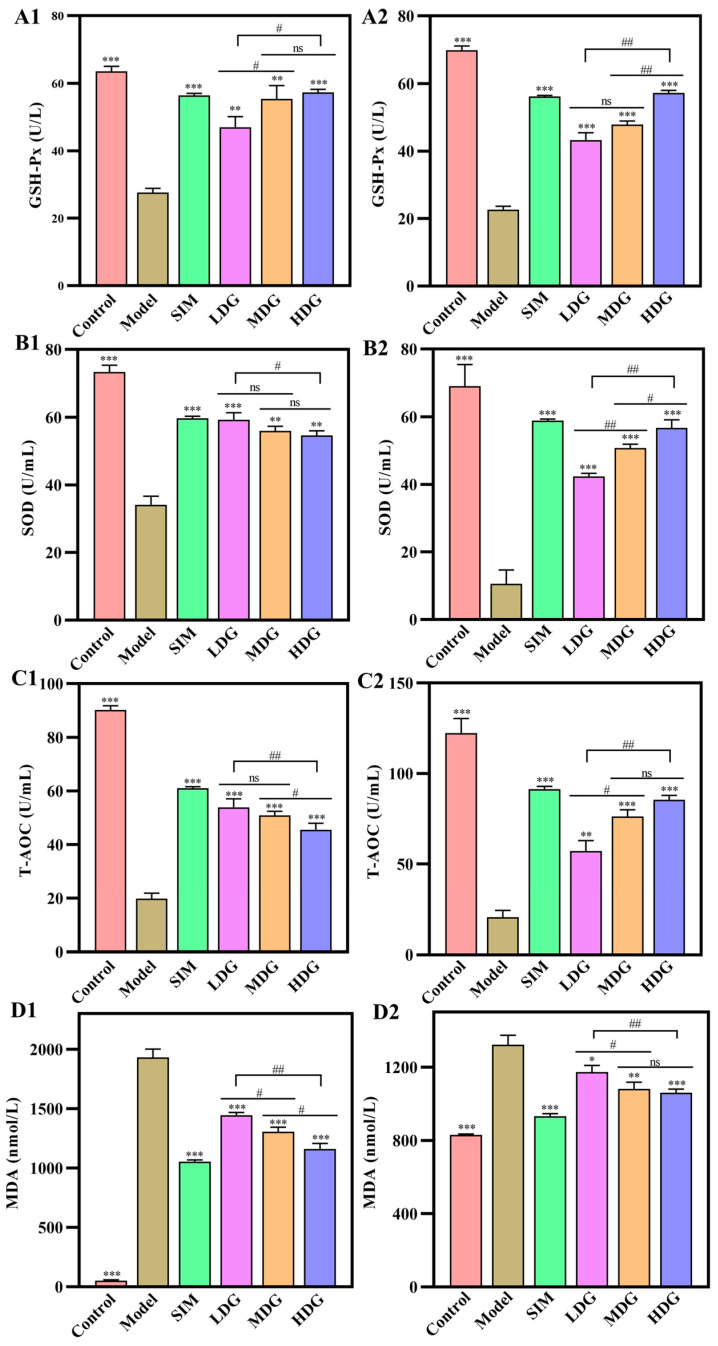
Oxidative stress indicator changes in the serum and liver after 30 days of macadamia oil intervention. (**A1**–**D1**) and (**A2**–**D2**) show the GSH-Px, SOD, T-AOC, and MDA levels in the serum and liver, respectively. ns denotes no significant differences between different letters at *p* < 0.05. *, **, and *** indicate *p* < 0.05, *p* < 0.01, and *p* < 0.001 versus the Model, respectively. # and ## denote significant differences between different letters at *p* < 0.05 and *p* < 0.01, respectively.

As depicted in Figure 5, the GSH-Px, SOD, and T-AOC levels in the serum and liver of the Model markedly declined relative to the Control, whereas the MDA levels showed an opposite trend. Moreover, MO-treated groups had significantly higher GSH-Px, SOD, and T-AOC levels and lower MDA levels in the serum and liver than those of the Model. In summary, MO treatment could increase the antioxidant activities of the serum and liver and lead to a decline in lipid oxidation in a dose-dependent manner. This was aligned with the study by Zhu et al. [30], which indicated that extra antioxidant supplementation could effectively prevent hyperlipidemia and atherosclerosis by improving lipid metabolism. Therefore, the hypolipidemic effect of MO might be closely associated with its antioxidant activities.

Figure 5A1–C1 showed that the GSH-Px, SOD, and T-AOC properties in the serum of the HDG were 2.09-fold, 1.60-fold, and 2.30-fold that of the Model values, respectively, while the properties of these indicators in the liver increased by 4.36-fold, 1.53-fold, and 3.13-fold, respectively. Meanwhile, these indicator levels of the HDG were almost similar to those of the SIM. In contrast, the MDA levels in the serum and liver of the HDG were reduced by 39.9% and 19.8% (Figure 5D1, Figure 5D2), respectively, compared with the Model. Therefore, MO played an essential role in alleviating oxidative stress through elevating activities of GSH-Px, SOD, and T-AOC and reducing MDA levels in the serum and liver, which further verified the hypolipidemic effects of MO on HepG2 cells reported by Shuai et al. [12].

### 3.5. The Effects of MO on Histological Changes in the Liver

**Figure 6:**
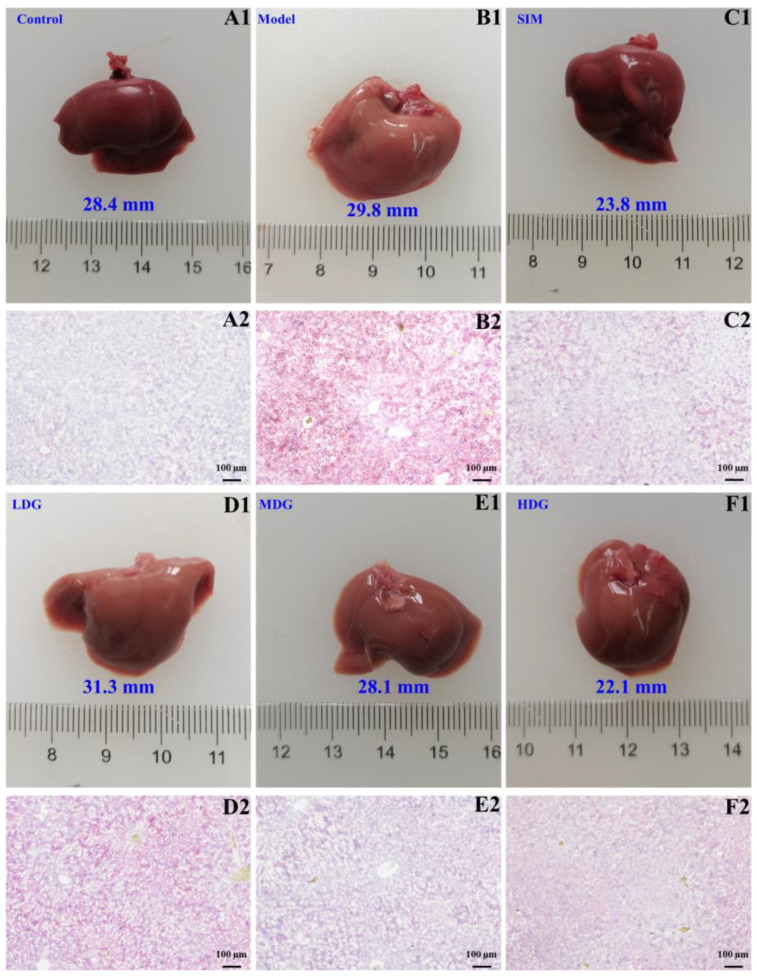
The effects of macadamia oil treatment on the histology and morphology of the livers of hyperlipidemic mice. (**A1**–**F1**) The morphologies of the livers of the Control, Model, SIM, LDG, MDG, and HDG after 30 days of intervention, respectively; (**A2**–**F2**) the photomicrographs of the hepatic tissues of the Control, Model, SIM, LDG, MDG, and HDG after Oil Red O staining (100×), respectively.

As shown in Figure 6B1, the Model liver appeared slightly white with a blunt and greasy edge, indicative of fatty liver, which was attributed to lipid accumulation in the liver tissue due to an HFD. In comparison with the Model, the Control liver (Figure 6A1) was rosy, the surface was smooth, and there was no swelling. Furthermore, the liver swelling in the SIM- and MO-treated groups (Figure 6C1–F1) showed different degrees of relief compared to the Model. And the liver features of the SIM were similar to those of the Control. Meanwhile, the liver appearance of the LDG, MDG, and HDG showed a dose-dependent improvement.

Meanwhile, the lipid deposition in liver tissues was evaluated using the Oil Red O staining method, which stained the lipid droplets in the liver tissues with a red color. As depicted in Figure 6A2, the liver cells in the Control appeared normal, exhibiting blue nuclei and no hepatic lipid droplet depositions. However, the deposition of hepatic lipid droplets was more obvious in the Model (Figure 6B2) compared to the Control. In the SIM- and MO-treated groups, lipid droplet accumulation was remarkably prevented in a dose-dependent manner (Figure 6C2–F2), whereas a large number of lipid droplets remained in the LDG. These results were consistent with the oxidative stress indicators observed in the liver (Section 3.4). As a result, MO supplementation was determined to effectively alleviate the deposition of hepatic lipids, thereby improving hyperlipidemia and preventing liver damage. Additionally, Han et al. [31] also reported a marked reduction in circular lipid droplets in the liver tissue of mice that were fed with flaxseed oil. The study by Cao et al. [32] demonstrated that medium-chain fatty acids could activate a CREBH-FGF21 axis to reduce hepatic lipid accumulation. Nevertheless, it was reported that the regulation of lipid metabolism in hepatocytes is closely related to the alleviating effects of oleic acid on autophagy dysfunction [33]. This provided a possible explanation for the improvement in fatty liver observed in the groups treated with MO (oleic acid content > 60%) (Figure 6D2–F2) in comparison with the Model (Figure 6B2).

### 3.6. The Effects of MO on Histological Changes in the Adipose

**Figure 7:**
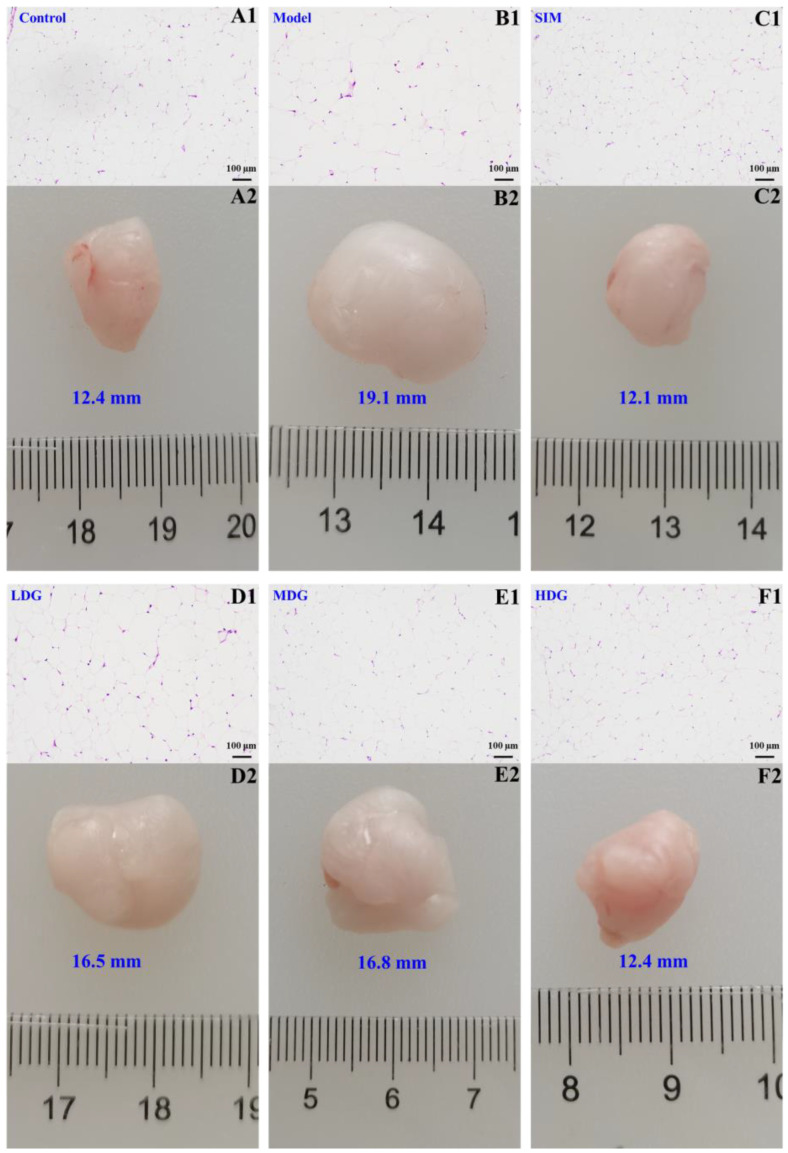
The effects of macadamia oil treatment on the histology and morphology of adipose tissues of hyperlipidemic mice. (**A1**–**F1**) The photomicrographs of the adipose tissues of the Control, Model, SIM, LDG, MDG, and HDG after HE staining (100×); (**A2**–**F2**) the morphologies of the adipose tissues of the Control, Model, SIM, LDG, MDG, and HDG after 30 days of intervention, respectively.

Generally, there are several typical symptoms of obesity, such as the expansion of adipose tissue and the enlargement of fat cells [34]. In the present study, the effects of MO on lipid accumulation were evaluated by the histology and morphology of the epididymis adipose tissues. As exhibited in Figure 7A2–F2, the adipocyte size in the Model was significantly larger than that in the Control, while the adipocyte size in both the SIM- and MO-treated groups exhibited a significant decrease compared with the Model. Meanwhile, high-dose MO treatment demonstrated a similar inhibitory effect on the growth of epididymis adipose compared to 10 mg/kg of BW of simvastatin.

To further confirm the preventive effects of MO on obesity, epididymis tissue HE staining was performed in this study. As depicted in Figure 7A1,B1, it was observed that the fat cell size in the SIM- and MO-treated groups was significantly smaller than that in the Model. Furthermore, MO treatment (Figure 7D1–F1) dramatically inhibited the enlargement of epididymis fat cells in a dose-dependent manner. Particularly, the epididymis adipose morphology of the HDG was similar to that of the SIM (Figure 7C1) and was generally close to that of the normal mice. However, what cannot be ignored is the adverse side effects of statins, including elevated liver function, myopathy, and new-onset diabetes [35]. Therefore, MO supplementation could significantly improve lipid accumulation in the liver without causing damage, indicating that MO might serve as an alternative to statins for improving lipid accumulation.

### 3.7. The Effects of MO on the AMPK/Nrf2 Pathway

**Figure 8:**
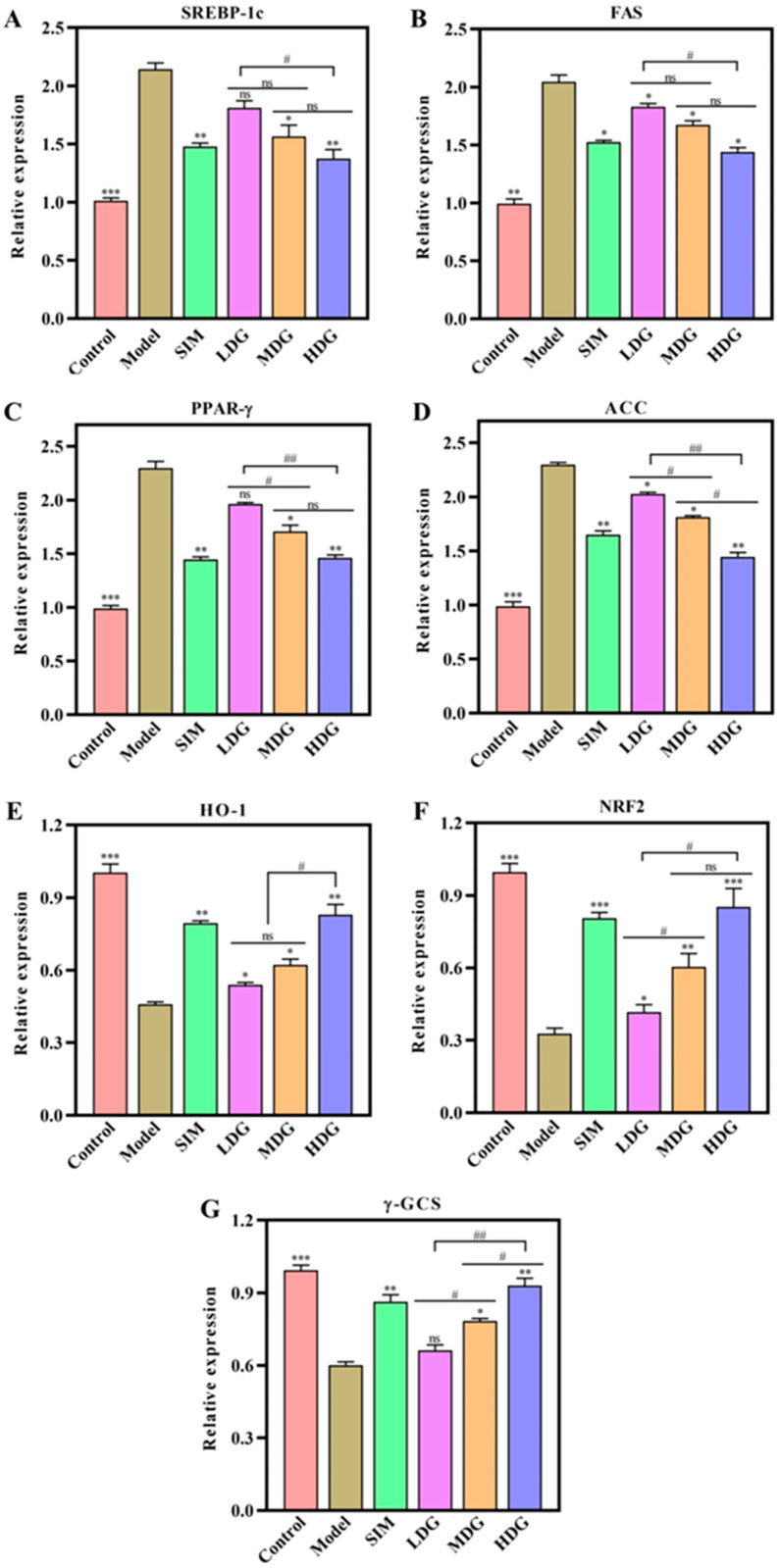
Changes in the expression levels of AMPK/Nrf2 pathway-related mRNA in the liver after 30 days of macadamia oil intervention. (**A**–**G**) represent SREBP-1c, FAS, PPAR-γ, ACC, HO-1, Nrf2, and γ-GCS, respectively. ns denotes no significant differences between different letters at *p* < 0.05. *, **, and *** indicate *p* < 0.05, *p* < 0.01, and *p* < 0.001 versus the Model, respectively. # and ## denote significant differences between different letters at *p* < 0.05 and *p* < 0.01, respectively.

**Figure 9:**
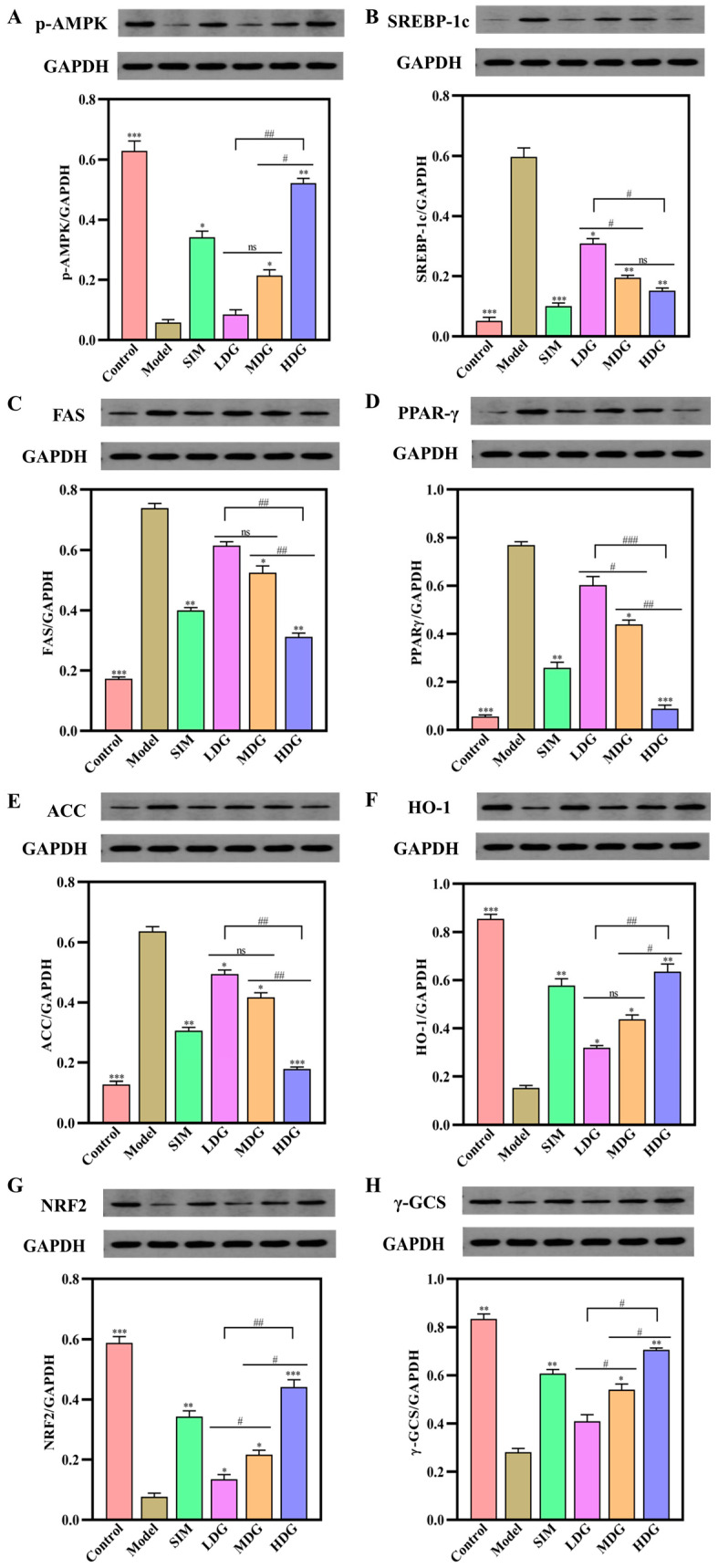
Changes in the expression levels of AMPK/Nrf2 pathway-related proteins in the liver after 30 days of macadamia oil intervention. (**A**–**H**) represent p-AMPK, SREBP-1c, FAS, PPAR-γ, ACC, HO-1, Nrf2, and γ-GCS, respectively. ns denotes no significant differences between different letters at *p* < 0.05. *, **, and *** indicate *p* < 0.05, *p* < 0.01, and *p* < 0.001 versus the Model, respectively. #, ##, and ### denote significant differences between different letters at *p* < 0.05, *p* < 0.01, and *p* < 0.001, respectively.

It was previously reported that the hypolipidemic effect of MO on lipid accumulation in HepG2 cells occurs through activating the AMPK/Nrf2 pathway [12]. This study was an extension of the previous research to further verify the lipid-lowering effects and mechanisms of MO in vivo. In this study, the effect of MO on the expression of AMPK/Nrf2 pathway-related genes and proteins was detected using qRT-PCR (Figure 8A–G) and WB (Figure 9A–H). As we all know, AMPK is essential in regulating lipid metabolism and energy balance, while the Nrf2 pathway is associated with oxidative stress [36]. In detail, Nrf2 is separated from the keap1 protein and phosphorylated when oxidative stress occurs. It then transfers to the nucleus, where it binds to antioxidant components and activates the Nrf2-induced antioxidant signaling pathway [37].

As exhibited in Figure 9A, the expression of p-AMPK was significantly lower in the HMG relative to the Control (*p* < 0.001). Furthermore, MO treatment increased the expression of p-AMPK in a dose-dependent manner, suggesting that the AMPK pathway was activated. Importantly, substantial evidence has confirmed that p-AMPK regulates liver lipid metabolism by inhibiting the expression of SREBP-1c and FAS [38]. As depicted in Figure 9B,C, the expression of SREBP-1c and FAS in the Model was significantly higher compared to the Control, while the expression in the SIM- and MO-treated groups decreased in a dose-dependent manner, indicating that MO supplementation might inhibit lipid accumulation of the liver. Meanwhile, the expression of ACC and PPAR-γ was also assessed in the present study (Figure 9D–E), which exhibited a significant increase in the HMG in comparison with the Control. Moreover, the effect of MO on the expression of ACC and PPAR-γ was in line with the SREBP-1c and FAS. These results further verified that MO supplementation prevented lipid accumulation by activating the AMPK pathway. Additionally, it inhibited the expression of SREBP-1c, FAS, ACC, and PPAR-γ, which aligned with the mRNA expression levels of genes related to the AMPK pathway (Figure 8A–D). These findings were aligned with the study by Chen et al. [5], which evaluated the lipid-lowering effect of hesperidin in HFD-induced HepG2 cells.

Figure 9G depicted that the Nrf2 level was downregulated in the Model relative to the Control, whereas MO treatment upregulated the Nrf2 level in a dose-dependent manner relative to the Model. Moreover, HO-1 and γ-GCS, which were downstream effectors of the Nrf2 pathway, were also analyzed to assess the effect of MO on preventing oxidative stress. As expected, MO supplementation significantly increased the expression of HO-1 (Figure 9F) and γ-GCS (Figure 9H), which aligned with the expression of Nrf2 (Figure 9G), indicating that the Nrf2 pathway was activated. Additionally, similar trends were observed in the SIM and mRNA expression results (Figure 8E–G). Accordingly, MO intervention significantly elevated the levels of Nrf2, HO-1, and γ-GCS, helping to reduce liver damage caused by oxidative stress.

**Figure 10:**
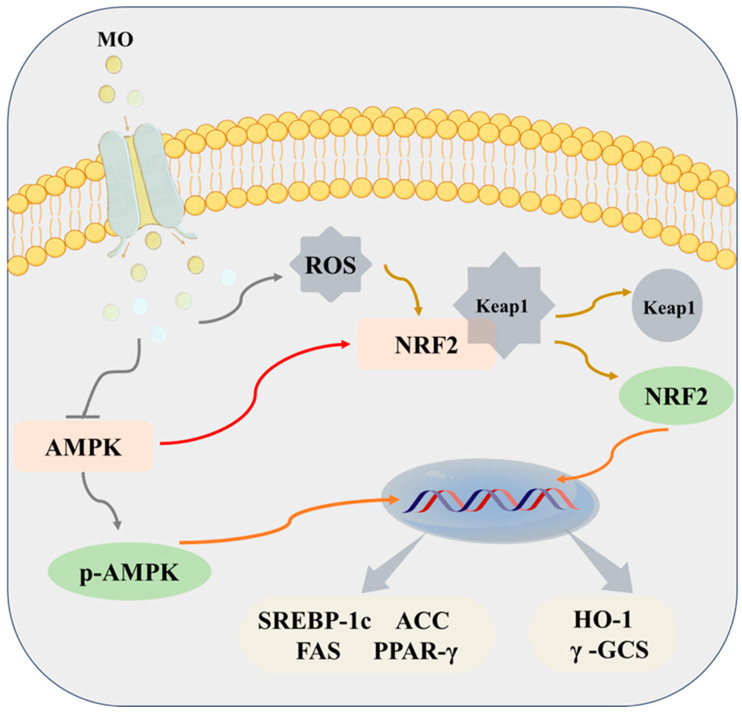
Schematic and potential mechanism for macadamia oil to relieve lipid accumulation.

As a crucial transcription factor, Nrf2 regulates the expression of oxidative stress-related genes, and its activity is regulated by AMPK [14]. Notably, accumulating evidence had confirmed that Nrf2 activation could markedly upregulate the protein expression of its downstream genes HO-1 and γ-GCS [39,40]. For instance, N1,N5-di-[(E)-p-coumaroyl]-spermidine separated from adlay could significantly increase the expression of HO-1 and γ-GCS to attenuate the oxidative stress of HepG2 cells [41]. Moreover, these results were also confirmed by Liu et al. [42], who evaluated the protective effect of *Paeonia lactiflora* petal flavonoid extract against H_2_O_2_-induced oxidative damage in BRL3A cells. Herein, MO could be used as a lipid-lowering oil to alleviate hyperlipidemia and oxidative stress by activating the AMPK/Nrf2 pathway, and its mechanism of action is shown in Figure 10.

## 4. Conclusions

In conclusion, this study highlighted the lipid regulation effects of MO on HFD-induced mice, including reduced BW and organ index values, lowered serum lipid, ALT, and AST levels, and oxidative stress relief. Furthermore, the lipid deposition of the liver and adipose tissue was significantly improved by MO administration. These improvements were attributed to MO supplementation activating the AMPK/Nrf2 pathway. Specifically, the suppressed effects of lipid accumulation of MO could be due to its ability to increase expression levels of p-AMPK and downregulate SREBP-1c, FAS, ACC, and PPAR-γ expression. Meanwhile, MO reduced oxidative stress by upregulating the expression of Nrf2 downstream genes HO-1 and γ-GCS. Nevertheless, MO, as a natural plant oil, requires further research to ensure its administration and dosage forms provide fundamental support for developing advanced functional foods or lipid-lowering medications.

## Figures and Tables

**Table 1:** Primer sequence of target genes.

Gene	Primer	Sequence (5′-3′)	PCR Products
Mus GAPDH	Forward	ATGGGTGTGAACCACGAGA	229 bp
	Reverse	CAGGGATGATGTTCTGGGCA	
Mus HO-1	Forward	GCAACAAGCAGAACCCAGTC	363 bp
	Reverse	TTCGGGAAGGTAAAAAAAGC	
Mus γ-GCS	Forward	CACATCTACCACGCAGTC	172 bp
	Reverse	GGTTGGGGTTTGTCCTC	
Mus Nrf2	Forward	CAGTGCTCCTATGCGTGAA	109 bp
	Reverse	GCGGCTTGAATGTTTGTCT	
Mus SREBP-1c	Forward	ACTTCTGGAGACATCGCAAAC	279 bp
	Reverse	GGTAGACAACAGCCGCATC	
Mus FAS	Forward	CCTGCCTCTGGTGCTTGCT	138 bp
	Reverse	GGGCCTCCTTGATATAATCCTT	
Mus PPAR-γ	Forward	ACCACTCGCATTCCTTT	264 bp
	Reverse	CACAGACTCGGCACTCA	
Mus ACC	Forward	CTGTATGAGAAAGGCTATG	149 bp
	Reverse	AAGAGGTTAGGGAAGTCA	

## Data Availability

The data presented in this study are available upon reasonable request from the corresponding author. The data are not publicly available due to privacy restrictions.
